# Promoting Drp1-Mediated Mitochondrial Division in Nickel Nanoparticles-Induced Reproductive Toxicity in GC-2 Cells

**DOI:** 10.3390/nano16010034

**Published:** 2025-12-25

**Authors:** Liya Qiao, Zhimin Tong, Yabing Xu, Chunliu Guan, Geyu Liang, Lu Kong

**Affiliations:** 1 Key Laboratory of Environmental Medicine and Engineering, Ministry of Education, School of Public Health, Southeast University, Nanjing 210009, China; lyqiao0126@126.com (L.Q.); 13585183112@163.com (Y.X.); clguan22@163.com (C.G.); 2 Kunshan Municipal Center for Disease Prevention and Control, Kunshan 215301, China; 79972002tzm@163.com

**Keywords:** Drp1, nickel nanoparticles, apoptosis, mitochondrial division, reproductive toxicity

## Abstract

Male reproductive disorders and declining fertility rates play an important role in birth rates, and their impact on future populations makes them one of the most serious public health issues of this century. Defects in spermatogenesis are the most common manifestation of male infertility, and exposure to environmental pollutants has been suggested as a potential cause. Nanomaterials, due to their unique physicochemical properties and widespread application, have raised growing concerns about their potential reproductive toxicity. Studies have shown that nickel nanoparticles (Ni NPs) have reproductive toxicity in male rats and mice, especially sperm damage. This study aimed to explore the male reproductive toxicity of Ni NPs and the role of mitochondrial fission in mouse spermatocytes (GC-2). Our results showed that Ni NPs induced the damage of mitochondrial structure and function in GC-2 cells and disrupted intramitochondrial homeostasis, thereby resulting in enhanced Dynamin-related protein 1(Drp1)-mediated mitochondrial fission and cell apoptosis, along with aggravated cytotoxicity and obvious reproductive toxicity. The mitochondrial division inhibitor 1(Mdivi-1) and lentiviral-transfected low expression of Dnm1l can significantly alleviate the germ cell toxicity caused by Ni NPs, suggesting a certain therapeutic effect. The novelty of this study lies in its systematic demonstration that Drp1-mediated mitochondrial division is a core pathogenic mechanism of Ni NP-induced male reproductive toxicity, and the validation of both pharmacological inhibition and genetic silencing as effective intervention strategies. Therefore, this study offers a reference for expanding the reproductive toxicity effect of Ni NPs and potential molecular mechanisms and provides an important basis for finding potential targets and treatment of Ni NPs.

## 1. Introduction

Worldwide infertility is a major and growing health problem influencing about 10–15% of all couples, and the incidence of male infertility accounts for approximately 50% of all infertile couples [1,2]. Male reproductive system disorders such as oligozoospermia, asthenozoospermia, and teratozoospermia directly decrease the probability of natural conception [3]. Endocrine disturbances, including decreased testosterone levels or abnormal gonadotropin secretion, may inhibit spermatogenesis and impair sexual function. Structural damage to the testes, such as varicocele or cryptorchidism, can disrupt the spermatogenic microenvironment, leading to spermatogenic dysfunction [4]. There are many different reasons for male infertility. Except for genetic causes in a small percentage of male infertile cases, most of them are seriously affected by external pollution, including environmental pollutants. Nickel, as an industrial pollutant and component of electronic waste, can enter the human body through various pathways. Mechanistically, its reproductive toxicity primarily includes oxidative stress damage, endocrine-disrupting effects, testicular histopathological alterations, and epigenetic dysregulation [5,6,7,8]. Understanding the male sperm defect may assist in the clinical management of male infertility, and sperm quality has been documented to be the main cause in 25–87% of male infertility cases.

With the development of nanotechnology, the wide application of nanomaterials might increase the chances of human exposure, making them become environmental pollutants [9,10,11]. Notably, nanoparticles may enter the reproductive system through multiple pathways. (i) Systemic circulation: nanoparticles can enter the bloodstream via inhaling, ingesting, or injecting into the body and subsequently distribute to reproductive organs [12,13]. (ii) Local direct exposure: In females, nanoparticles from hygiene products or medications (e.g., vaginal applications) may permeate through mucosal membranes into the reproductive tract [14,15]. (iii) Barrier penetration: nanoparticles can bypass protective barriers via passive diffusion or active transport. For instance, crossing the blood–testis barrier may disrupt spermatogenesis, while traversing the placental barrier enables maternal–fetal transfer during pregnancy [16,17]. After entering the human body, nanomaterials are more likely to pass through the blood–testis barrier and placental barrier than micron-sized substances due to their ultra-small particle size, and then accumulate in reproductive organs, resulting in reproductive and developmental toxicity, as demonstrated by higher reproductive toxic effects in a variety of nanomaterials such as nano-titanium dioxide, nano-silver, nano-nickel, etc. [18,19]. Notably, these nanoparticles can not only penetrate germ cells through membrane interstices or endocytosis but also disrupt tight junction proteins in the blood–testis barrier, thus entering seminiferous tubules and directly interacting with spermatogonia [20]. Within cells, nanoparticles generate reactive oxygen species (ROS), consequently damaging mitochondrial function and leading to sperm DNA fragmentation [21]. As demonstrated in a previous study, carbon-based nanoparticles may alter DNA methylation patterns, affecting gene expression in germ cells and even inducing transgenerational epigenetic effects [22]. Additionally, certain nanoparticles (e.g., ZnO NPs) can interfere with the hypothalamic–pituitary–gonadal axis, reduce testosterone levels, and inhibit oocyte maturation [23].

More recently, nickel nanoparticles (Ni NPs) with high global production have been widely applied in numerous fields, especially in the field of biomedicine due to the characteristics of targeting and their direct entry into the human body [24,25,26]. Additionally, Ni NPs exhibit diverse applications in daily life and technology owing to their unique properties, such as high reactivity, large specific surface area, strong magnetism, and low melting point [27]. In the energy sector, they serve as high-efficiency catalysts in hydrogen fuel cells, lithium-ion battery electrode materials, and solar cell components, enhancing energy conversion efficiency [28,29,30,31,32]. In healthcare, they enable targeted tumor therapy through magnetic hyperthermia and are utilized in developing antibacterial coatings or implant materials leveraging their antimicrobial properties [33,34,35]. Accumulating evidence has demonstrated that Ni NPs synthesized via a green synthesis approach are more environmentally friendly in terms of their scenarios, such as photocatalytic degradation of pollutants and self-cleaning surface coatings [36,37,38,39,40,41]. In fact, there are different ways in which human beings are exposed to Ni NPs. Except for high-concentration occupational exposure that may directly induce respiratory diseases such as pulmonary inflammation and fibrosis in industrial production, environmental Ni NPs can also bioaccumulate through the food chain, and chronic low-dose exposure may disrupt endocrine function, leading to germ cell apoptosis and testicular damage [8]. Accordingly, experts and scholars have been paying close attention to the adverse effects of Ni NPs on reproductive health-related problems.

With the in-depth study of Ni NP-induced toxicity, an increasing number of recent reports have provided evidence that exposure to Ni NPs has reproductive and developmental toxicity, such as the obvious symptoms of lower sperm quality, declined hatchability of fertilized eggs, prolonged hatching time, and reduced size of embryos, consequently causing abnormal offspring development in model animals [42,43,44,45].

Our recent research has documented that Ni NPs can damage the testicular structure, leading to a destruction of the blood–testis barrier, a decrease in sperm motility, and an increase in sperm malformation. Similarly, both rodent models and Caenorhabditis elegans exhibited marked reduction in serum reproductive hormone levels following Ni NPs administration. Intriguingly, comparative analyses revealed that the male reproductive system exhibits heightened sensitivity to Ni NPs compared to the female counterpart [19,46,47], and the exact reasons should be further studied. A previous study suggested that Ni NPs exerted reproductive toxicity through oxidative stress, lipid peroxidation, DNA breakage, and the alteration of mitochondrial membrane potential (MMP) [45]. These specific mechanisms are supported by multiple cellular structures, including cell membrane damage, mitochondrial dysfunction, endoplasmic reticulum stress, and lysosomal rupture [48,49,50]. Our study revealed that Ni NP-induced reproductive toxicity in rat primary spermatogenic cells is mechanistically associated with programmed cell death, involving both the LOC102551356/IGF-BP3/p53 signaling cascade and mitochondrial-dependent apoptotic mechanisms [51]. In particular, the pathological changes and the ultrastructure of the testicular tissue were confirmed in the structural damage, vacuolization, and increased number of mitochondria, etc., which have been recognized by peers at home and abroad and documented in the Journal Chemosphere [47]. Furthermore, some previous studies have implicated mitochondria as playing a central and early role in cell apoptosis [52,53]. Consequently, it can be inferred that mitochondrial serves as a primary site for Ni NP-induced toxicity, leading to disruptions in reproductive system architecture and physiological functions, ultimately triggering apoptotic processes in germ cells.

As one of the most important organelles in cells, mitochondria, termed the powerhouse of the cell, produce ATP through oxidative phosphorylation to maintain normal physiological functions of cells, and are also the center of cell metabolism and signal transduction that participate in biological processes such as calcium ion homeostasis and regulate various cellular functions [54] such as spermatogenic cell apoptosis, etc. Theoretically, the mechanism of male infertility underlying the spermatogenic process, together with its influencing factors, is an important theoretical basis for exploring new methods of male infertility. Mitochondria are dynamic organelles, constantly undergoing division and fission to maintain a balance between them. Several studies have suggested that mitochondrial division is presented as an intermediate link in the process of cell apoptosis [55], and excessive division resulting in fragmented and dysfunctional mitochondria has been observed in numerous diseases [56,57]. As far as we know, the mitochondrial division has not been illustrated in the impairment of spermatogenic cells in exposure to Ni NPs; therefore, we focus on the role of mitochondrial division in the reproductive toxicity of this study.

Moreover, a previous study [58] has verified that mitochondrial division is closely related to dynamin-related protein 1(Drp1), and the abnormal mitochondrial division mediated by Drp1 is highly associated with apoptosis. This is also confirmed by our previous study [59] that mitochondrial division factor Drp1 was upregulated in mouse spermatogonia in exposure to Ni NPs, along with the elevation of mitochondrial reactive oxygen species (mtROS), which plays an essential biological process in cell apoptosis. However, it was also unclear whether Ni NPs cause the impairment of spermatogenic cells through mtROS, as well as the specific cellular and molecular mechanisms. Consequently, it is necessary to further explore the role of mitochondrial division and mtROS in the Ni NP-induced apoptosis of spermatogenic cells.

Herein, GC-2 cells from the spermatogonia cell line of the mouse were used to investigate the role of mitochondrial division in regulating the apoptosis of germ cells induced by Ni NPs, which was further confirmed by both the inhibitor of mitochondrial division and lentivirus transfection. The results demonstrated that Ni NPs significantly reduced the viability, induced morphological damage, and triggered excessive apoptosis of GC-2 cells, thereby exhibiting distinct germ cell toxicity. Interestingly, mitochondrial division inhibitor 1(Mdivi-1) effectively alleviated the Ni NP-induced decline in cell viability, increased apoptosis rate, accumulation of ROS and MtROS, as well as the reduction in ATP levels and MMP. Additionally, Dnm1l knockdown (Dnm1l-KD) mitigated the cellular toxicity and mitochondrial damage caused by Ni NPs. And the regulatory mechanism of the mtROS/Drp1 signal axis was also elucidated in the Ni NP-induced male reproductive injury.

As a result, except for serving as a reference for further exploring the potential mechanism underlying the Ni NP-induced male reproductive toxicity, our current study may provide a scientific basis for the identification of potential targets of Ni NPs treatment and the protection of reproductive toxicity of environmental and occupational exposure to Ni NPs.

## 2. Materials and Methods

### 2.1. Cell Line

GC-2 cells, a mouse pachytene spermatocyte-derived cell line, were donated from the School of Public Health, Nanjing Medical University (Nanjing, China). The cells were cultured with Dulbecco’s modified Eagle’s medium (DMEM; Gibco; Waltham, MA, USA) supplemented with 10% fetal bovine serum and 1% penicillin at 37 °C in a humidified environment containing 5% CO_2_.

### 2.2. Ni NPs

Ni NPs (product number: FNiN-80; purity: 99%; surface area: no less than 8 m^2^/g; bulk density: 0.06–0.8 g/cm^3^; average size: 90 nm) were black powder and purchased from Nano Science and Kunshan City Miyou Technology Co., Ltd., Kunshan, China Company [51,60,61].

### 2.3. Characterization and Preparation of Ni NPs Suspension

Characterization of Ni NPs was assessed by scanning electron microscopy (SEM, JEOL, Tokyo, Japan) and transmission electron microscopy (TEM, JEOL, Tokyo, Japan), and the Ni NP suspension was prepared according to our previous studies [19,62].

### 2.4. In Vitro Cytotoxic Assay

GC-2 cells were first seeded into a 96-well plate at a density of 8 × 10^3^ cells/100 μL per well, with at least three replicate wells for each sample in a Carbon Dioxide Incubator (HERAcell 150I, Thermo, USA). After culturing for 24 h, the cells were further incubated with different concentrations of Ni NPs for another 24 h. Following the addition of the CCK-8 solution (Nanjing Vazyme Biotech Co., Ltd., A311-01, Nanjing, China) to each well, the cells were incubated in the dark for 1.5 h, and then the optical density (OD) was measured at 450 nm on an automatic microplate reader. After repeating the experiment three times, the cell viability (%) [=(OD_experimental group_ − OD_blank_)/(OD_control group_ − OD_blank_) × 100%] was calculated, and the half inhibition concentration (IC50) fitted curve was determined by GraphPad Prism 8.0.2 software.

### 2.5. Observation of Cell Morphology

GC-2 cells were seeded into a 6-well plate at a density of 1.8 × 10^5^ cells/1 mL per well in a CO_2_ incubator. After culturing for 24 h, the cells were further incubated with Ni NPs at concentrations of 0, 25, 50, and 100 μg/mL for 24 h. Thereafter, the changes in cell morphology were observed by the Axiovert A1 inverted fluorescence microscopy (Axiovert A1, ZEISS, Oberkochen, Germany).

### 2.6. Observation Cell Ultrastructure

GC-2 cells were seeded into a 10 cm plate at a density of 1.8 × 10^5^ cells/5 mL per plate in a CO_2_ incubator. After culturing for 24 h, the cells were further incubated with or without 100 μg/mL Ni NPs for 24 h. Following trypsin digestion and centrifugation, the collected cells were fixed with 2.5% glutaraldehyde for 2 h. After rinsing with cold PBS several times, the cells were fixed with 1% osmic acid fixative for 2 h. Later, the steps of dehydration, soaking, embedding, and ultra-thin sectioning (50–70 nm) were executed sequentially. Finally, the cells were stained with uranyl acetate and lead citrate on copper mesh for the observation of cell ultrastructure by TEM (H-7650C, Hitachi, Japan).

### 2.7. Analysis of Cell Apoptosis

GC-2 cells were seeded into a 6-well plate at a density of 1.8 × 10^5^ cells/1 mL per well in a CO_2_ incubator. After culturing for 24 h, the cells were further incubated with Ni NPs at concentrations of 0, 25, 50, and 100 μg/mL for 24 h. Following trypsinization with EDTA-free enzyme, the harvested cells were resuspended in 500 μL of binding solution. Subsequent staining with 5 μL FITC and 5 μL PI was performed under light-protected conditions for 15 min. Thereafter, cell apoptosis was detected by flow cytometry (FACSCalibur, BD Biosciences, San Jose, CA,, USA), and the apoptosis rate was analyzed by FlowJo_V10 software.

### 2.8. Analysis of Mitochondrial Morphology

GC-2 cells were seeded into a 15 mm confocal dish at a density of 6 × 10^4^/mL per dish, cultured for 24 h, and followed with incubation of 0, 25, 50, and 100 μg/mL Ni NPs for 24 h. Simultaneously, the Mito Tracker Red CMXRos powder was dissolved in DMSO, and a 1 mM stock solution was diluted to a 100 nM work solution with an additional complete culture solution. Thereafter, a 1 mL work solution was added to each dish and incubated in the dark at 37 °C for 40 min. After rinsing with PBS twice, the cells were diluted with a pre-warmed complete culture medium to observe cell mitochondrial morphology (Ex = 579 nm, Em = 599 nm) by confocal laser scanning microscopy (FV3000, Olympus, Tokyo, Japan).

### 2.9. Detection of Reactive Oxygen Species (ROS)

GC-2 cells were seeded into a 6-well plate at a density of 1.8 × 10^5^ cells/1 mL per well in a CO_2_ incubator. After culturing for 24 h, the cells were further incubated with Ni NPs at concentrations of 0, 25, 50, and 100 μg/mL for 24 h. Simultaneously, a stock solution of the DCFH-DA probe was diluted to a 10 µM working solution with a serum-free culture medium at 1:1000. Then, 1 mL of the working solution was added to each well and incubated in the dark at 37 °C for 40 min. After rinsing with PBS three times, the inverted fluorescence microscopy (Axiovert A1, ZEISS, Oberkochen, Germany) was used to observe intracellular fluorescence intensity (Ex = 488 nm, Em = 525 nm) of GC-2 cells, and then Image J (1.52a) software was used to analyze the fluorescence intensity.

### 2.10. Determination of Mitochondrial Reactive Oxygen Species (MtROS)

GC-2 cells were plated in 12-well culture dishes at a density of 6 × 10^4^ cells per well in 500 μL medium and maintained for 24 h. After treatment with 0–100 μg/mL Ni NP concentrations, the cultures were continued for an additional 24 h period. The MitoSOX Red Mitochondrial Superoxide Indicator (5 mM stock) was prepared in PBS to achieve a 5 μM working concentration. Subsequently, cellular specimens were stained with 1 mL of working solution per well under standard culture conditions for 10 min. Mitochondrial reactive oxygen species (MtROS) fluorescence signals (Ex = 510 nm, Em = 580 nm) were imaged using an inverted fluorescence microscope (Axiovert A1, ZEISS, Oberkochen, Germany).

### 2.11. Measurement of Adenosine Triphosphate (ATP)

GC-2 cells were seeded into a 6-well plate at a density of 1.8 × 10^5^/1 mL per well, and cultured for 24 h. After exposure to 0, 25, 50, and 100 μg/mL Ni NPs for 24 h, the cells were rinsed with PBS three times and lysed with 200 µL ATP assay lysate per well. After centrifugation at 4 °C for 5 min at 12,000 round per minute (rpm), the supernatant was taken for ATP detection. Meanwhile, the ATP standard solution was diluted with ATP assay lysis buffer to form different concentration gradients for standard curve determination. Following the additional 100 µL ATP work solution in each well of the black 96-well plate at room temperature for 3–5 min, a 20 µL sample to be tested was added and mixed to measure the relative light unit on a multifunctional microplate reader (Tristar 5 LB942, Berthold, Bad Wildbad, Germany). Finally, the ATP content in each sample was obtained from the standard curve.

### 2.12. Measurement of Mitochondrial Membrane Potential (MMP)

GC-2 cells were seeded into a 6-well plate at a density of 1.8 × 10^5^/1 mL per well and cultured for 24 h. After culturing with 0, 25, 50, and 100 μg/mL Ni NPs for another 24 h, the cells were further incubated with complete medium and 1 mL JC-1 per well at 37 °C for 1 h. After rinsing with 4 °C JC-1 staining buffer twice, the cells with JC-1 monomer (Ex = 514 nm, Em = 529 nm) and JC-1 polymer (Ex = 585 nm, Em = 590 nm) were observed under an inverted fluorescence microscopy (Axiovert A1, ZEISS, Oberkochen, Germany), and then the fluorescence intensity was analyzed by Image J (1.52a) software.

### 2.13. Analysis of Western Blots

Western blot analysis was performed to assess the expression levels of various proteins across all experimental groups. Total proteins were isolated from GC-2 cells, which were then thoroughly lysed by the addition of RIPA lysis buffer (Beyotime Biotechnology, China) supplemented with protease and phospholipase inhibitors. Following centrifugation at 14,000 revolutions per minute for 12 min, the resulting supernatant was collected to measure protein concentrations via the BCA method (Beyotime Biotechnology, Catalog No. P0009, Shanghai, China). After quantification, the protein samples were heat-denatured in boiling water (100 °C) for 5 min and preserved at −80 °C for subsequent use.

In subsequent steps, the denatured proteins were separated using SDS-PAGE electrophoresis and transferred onto polyvinylidene fluoride (PVDF) membranes. The membranes were first blocked with 5% skimmed milk for 2.5 h, then incubated with primary antibodies (Boster, Pleasanton, CA, USA) diluted in 5% bovine serum albumin at 4 °C on a shaking platform for 12 h. This was followed by incubation with secondary antibodies (dilution ratio 1:5000; Boster, Pleasanton, CA, USA) for 2 h. After three rounds of washing with TBST buffer, the membranes were subjected to exposure in a chemiluminescent imaging system (Thermo Fisher Scientific, Waltham, MA, USA) to detect protein expression. Finally, Image J software was utilized to analyze the gray values of the protein bands, enabling the determination of relative protein expression levels.

### 2.14. Optimal Conditions for Mdivi-1 and Dnm1l and Their Reverse Effects on the Ni NPs-Induced Toxicity

To further explore the role of mitochondrial division in Ni NP-induced reproductive toxicity, GC-2 cell was used as a cell model and then exposed to 100 µg/mL Ni NPs with 0, 5, 10, 15, 20, and 25 μM Mdivi-1 for 24 h, and then cell viability was detected to identify the most optimal Mdivi-1 concentration. Thereafter, the GC-2 cells were separately cultured with DMEM (control group), 100 μg/mL Ni NPs (Ni NPs group), optimal Mdivi-1 (Mdivi-1 group), and 100 μg/mL Ni NPs + optimal Mdivi-1 (Ni NPs + Mdivi-1 group) for the subsequent experimental study.

Next, the GC-2 cells were infected with 0, 10, 20, and 40 MOI of lentivirus for 72 h. The proportion of fluorescent cells was observed, and the expression level of Drp1 protein was determined by Western blot assay. Then, the stably transfected cell lines of DNM1L-KD and DNM1L-NC of GC-2 cells were screened. Thereafter, the cells were separately cultured with DMEM (control group), 100 μg/mL Ni NPs (Ni NPs group), 100 μg/mL Ni NPs + DNM1L-KD (DNM1L-KD group), and 100 μg/mL Ni NPs + DNM1L-NC (DNM1L-NC group) for subsequent experimental study.

### 2.15. Statistical Analysis

Experimental data were expressed as mean ± standard deviation (Mean ± SD). One-way ANOVA was used for the comparison of means between multiple groups, and the LSD method was used for pairwise comparison. If the variance was not equal, the Welch test was used instead for overall mean comparison, and then the Dunnett T3 test was used for pairwise comparison. Statistical software SPSS 26.0 was applied to the statistical analysis of all data, and a *p*-value less than 0.05 indicated that the difference was statistically significant.

## 3. Results and Discussions

### 3.1. Toxicity of Ni NPs on Reproductive Cells

#### 3.1.1. Characterization of Ni NPs

The characterization of Ni NPs has been reported in detail in our previous experimental studies [19,46,63]. The detailed information can be tracked in the Supplementary Material (Figure S1).

#### 3.1.2. Effect of Ni NPs on Cytotoxicity, Morphology, and Apoptosis

**Figure 1:**
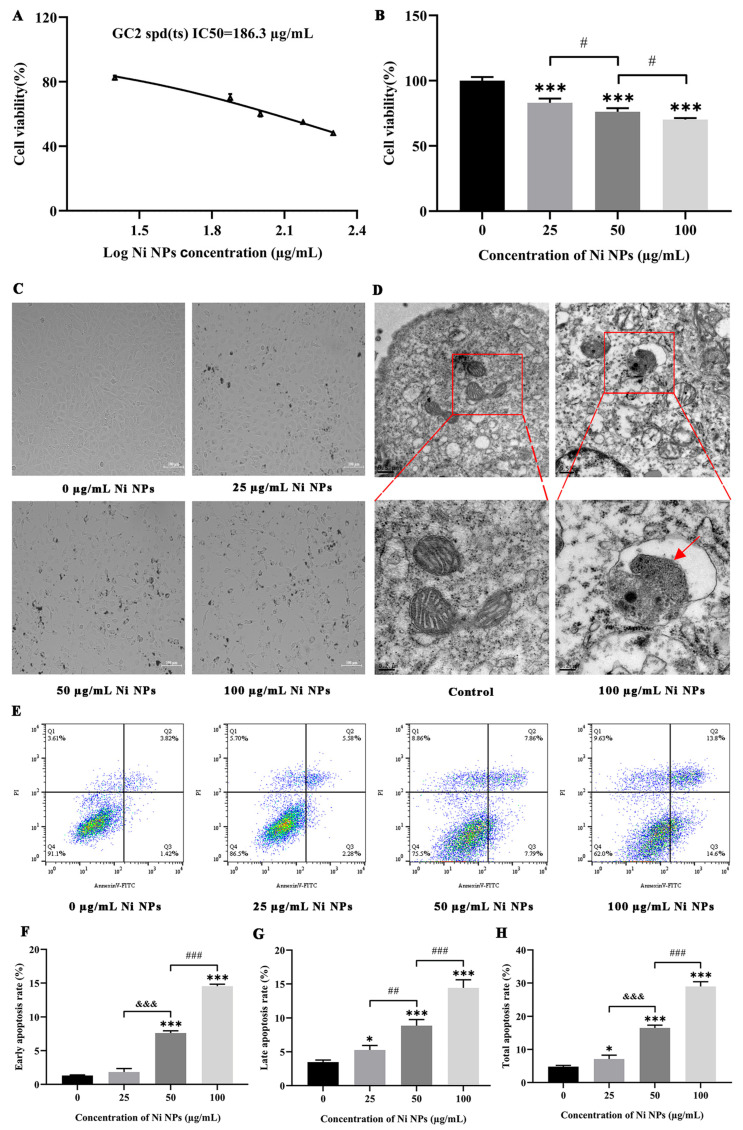
Cytotoxic effect of Ni NPs on GC-2 cells. Notes: (**A**) Curve-fitting of IC50 of GC-2 cells treated with different concentrations of Ni NPs for 24 h. (**B**) The viability of GC-2 cells treated with different concentrations of Ni NPs for 24 h. (**C**) Morphology of GC-2 cells treated with different concentrations of Ni NPs for 24 h under light microscopy (100×). (**D**) Representative images of the ultrastructure of GC-2 cells treated with or without 100 μg/mL Ni NPs for 24 h under transmission electron microscopy (Scale bar: 0.5 µm for the above images and 0.2 µm for the local enlarged images of the above). (**E**–**H**) Dot plot images (**E**), and histogram of early (**F**), late (**G**), and total apoptosis rate (H) in GC-2 cells. * *p* < 0.05, *** *p* < 0.001, compared to the control group. ^#^ *p* < 0.05, ^##^ *p* < 0.01, ^&&&^ *p* < 0.001, compared to the 25 μg/mL Ni NP group. ^###^ *p* < 0.001, compared to the 50 μg/mL Ni NP group.

GC-2 cells were used as experimental subjects to investigate the cytotoxic effect of Ni NPs. The IC50 of GC-2 cells treated with Ni NPs was 186.3 µg/mL (Figure 1A). Compared to the control group (0 µg/mL Ni NPs), the cell activity of different Ni NP groups decreased in a dose-dependent manner (*p* < 0.05) (Figure 1B), indicating that Ni NPs significantly inhibit the cell activity of GC-2 cells. Under light microscopy, the numerical density of GC-2 cells decreased, and the number of suspension cells increased with the increasing concentration of Ni NPs, accompanied by an increasing number of round cells and the large intercellular gap in the higher Ni NP groups compared to the control group (Figure 1C). Under the TEM, the cell ultrastructure showed abnormal mitochondrial morphology, such as a dramatic reduction in mitochondrial cristae, mitochondrial damage, and disorganized and aberrant cristae formation (Figure 1D), especially the appearance of mitochondrial autophagic vesicles (red arrows) in the 100 µg/mL Ni NP group compared to the control group. Based on these observations, it can be concluded that Ni NPs really have toxic effects. Moreover, the flow cytometry analysis showed that compared to the control group, the late and total apoptosis rates of cells in different Ni NP groups increased in a dose-dependent manner, and the early apoptosis rate of cells also increased in the 50 and 100 µg/mL Ni NP groups (*p* < 0.05), but not in the 25 µg/mL Ni NP group (Figure 1E). These results suggest that exposure to Ni NPs promotes the apoptosis of GC-2 cells.

#### 3.1.3. Effects of Ni NPs on Oxidative Stress

**Figure 2:**
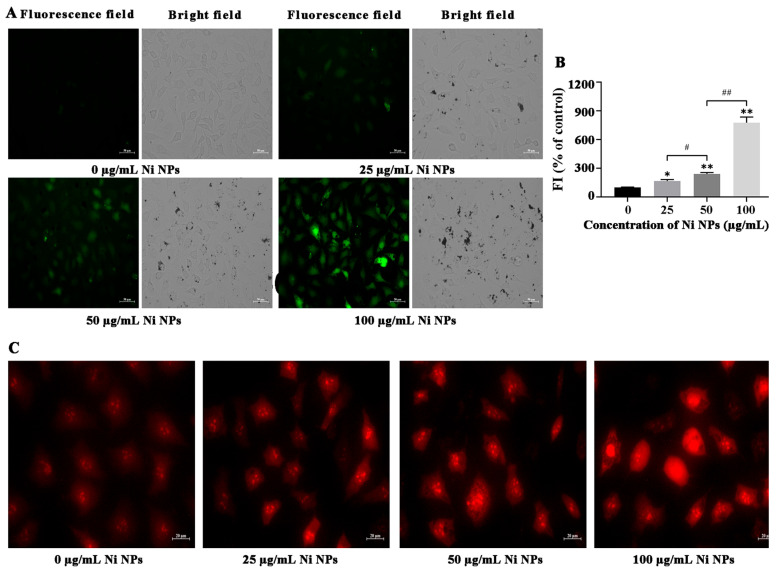
Oxidative stress responses of Ni NPs in GC-2 cells. Notes: (**A**) Representative fluorescence images of green ROS marker (DCFH-DA) in GC-2 cells treated with different concentrations of Ni NPs for 24 h. (**B**) Relative green fluorescence intensity of GC-2 cells. (**C**) Representative fluorescence images of red MtROS marker in GC-2 cells treated with different concentrations of Ni NPs for 24 h. * *p* < 0.05, ** *p* < 0.01, compared to the control group; ^#^ *p* < 0.05, compared to the 25 μg/mL Ni NP group; ^##^ *p* < 0.01, compared to the 50 μg/mL Ni NP group.

ROS and MtROS were performed to investigate the oxidative stress responses of Ni NPs in GC-2 cells. Under the inverted fluorescence microscopy, the number of GC-2 cells with green fluorescence intensity increased with the increasing concentration of Ni NP (Figure 2A), and there was a significant difference compared to the control group (*p* < 0.05) (Figure 2B), indicating that exposure to Ni NPs dose-dependently induces the production and accumulation of ROS in GC-2 cells. Similarly, GC-2 cells with red fluorescence intensity increased with the increasing concentration of Ni NPs (Figure 2C), suggesting that exposure to Ni NPs also induces an increase in MtROS in GC-2 cells.

#### 3.1.4. Effects of Ni NPs on Mitochondrial Structure and Function

**Figure 3:**
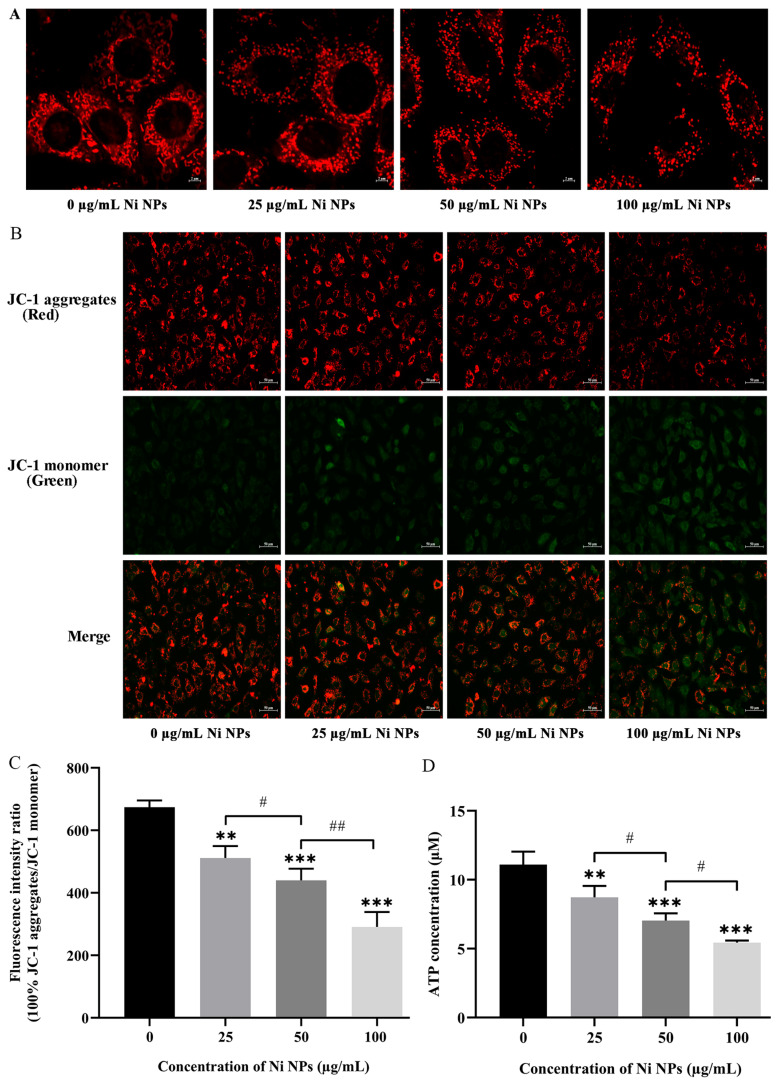
Effects of Ni NPs on mitochondrial structure, MMP, and ATP in GC-2 cells. Notes: (**A**) Representative mitochondrial morphology of GC-2 cells treated with different concentrations of Ni NPs for 24 h under confocal laser scanning microscopy (600×). (**B**) Representative fluorescence images of the MMP marker (JC-1) in GC-2 cells treated with different concentrations of Ni NPs for 24 h under inverted fluorescence microscopy. (**C**) Relative fluorescence intensity of GC-2 cells. (**D**) ATP content of GC-2 cells treated with different concentrations of Ni NPs for 24 h. ** *p* < 0.01, *** *p* < 0.001, compared to the control group; ^#^ *p* < 0.05, compared to the 25 μg/mL Ni NP group; ^##^ *p* < 0.01, compared to the 50 μg/mL Ni NP group.

To better understand the effect of Ni NPs on cellular mitochondria, mitochondrial morphology was first observed. Under laser confocal microscopy, Ni NPs impaired mitochondrial morphology and enhanced mitochondrial division compared to the control group (Figure 3A). Next, the MMP level and ATP content of the cells were also measured, and the results showed that the red fluorescence (JC-1 polymer) of the cells in the Ni NPs group gradually weakened, while the green fluorescence (JC-1 monomer) gradually brightened with the increasing concentration of Ni NPs (Figure 3B). Interestingly, the fluorescence intensity analysis showed that the ratio of red fluorescence to green fluorescence in the Ni NP groups dose-dependently decreased compared to the control group (*p* < 0.05) (Figure 3C). Similarly, Ni NPs decreased the MMP level and ATP content in a dose-dependent manner (Figure 3D), consequently influencing the cell growth of GC-2 cells.

#### 3.1.5. Effects of Ni NPs on the Expression of Mitochondria- and Apoptosis-Related Proteins

**Figure 4:**
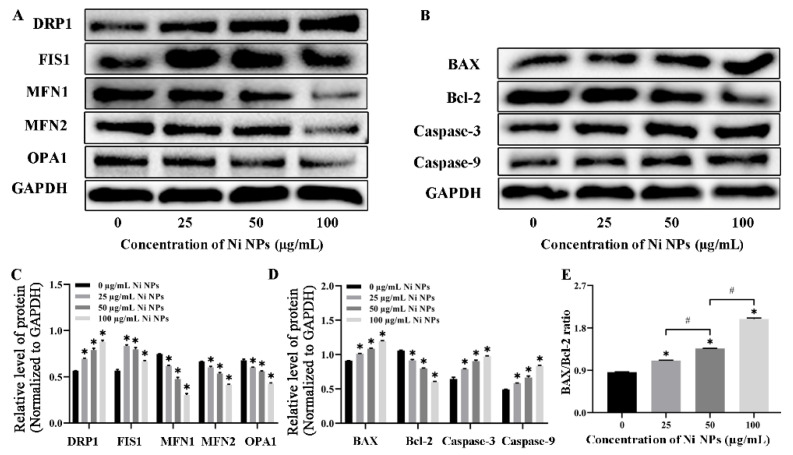
Expression of Ni NP-induced mitochondria- and apoptosis-related proteins in GC-2 cells. Notes: (**A**) Representative blot of mitochondria-related proteins in GC-2 cells treated with different concentrations of Ni NPs for 24 h. (**B**) Relative expression levels of the mitochondria-related proteins. (**C**) Representative blot of apoptotic proteins in GC-2 cells treated with different concentrations of Ni NPs for 24 h. (**D**) Relative expression levels of the apoptotic proteins. (**E**) Ratio of Bax/Bcl-2 in GC-2 cells. * *p* < 0.05, compared to the control group; ^#^ *p* < 0.05, compared to the Ni NP group.

To further analyze the detrimental effect of Ni NPs on the mitochondria of germ cells and cell apoptosis, we investigated the expression of mitochondria- and apoptosis-related proteins. Compared to the control group, the expression of apoptotic proteins Bax, Caspase-3, and Caspase-9 (Figure 4C,D) and mitochondrial splitting proteins Drp1 and Fis1 (Figure 4A,B) significantly increased in different concentrations of Ni NP groups (*p* < 0.05), whereas the expression of anti-apoptotic protein Bcl-2 (Figure 4C,D) and mitochondrial fission proteins Mfn1, Mfn2, and Opa1 remarkably decreased (*p* < 0.05) (Figure 4A,B). In addition, the ratio of Bax/Bcl-2 was significantly higher than that of the control group (*p* < 0.05). All these results suggest that exposure to Ni NPs increases mitochondrial division and inhibits mitochondrial fission in GC-2 cells, thereby leading to an accelerated germ cell apoptosis.

The decline in fertility caused by male reproductive disorders has become a worldwide reproductive health problem, and exposure to environmental pollutants is considered a potential etiology [64]. In general, the most common organic cause of male infertility is spermatogenesis defects, accounting for 20–25%. As a complex and continuous process, the stages of spermatogenesis include spermatogonia proliferation, spermatocyte meiosis, and differentiation of sperm cells into mature sperm [65]. Among mouse spermatocytes, the commercially available GC-2 cell, which was derived from a 6-week mouse testis, represents a stage between preleptotene spermatocytes and round spermatocytes [66]. In this study, GC-2 cell was used as a sperm cell model to explore the regulation mechanism underlying the potential male reproductive toxicity of Ni NPs.

Recently, increasing evidence has shown that apoptosis, a process of programmed cell death, plays an indispensable role in spermatogenesis [67] to maintain a proper balance between the germ cells and supporting sertoli cells. In this study, Ni NPs reduced cell viability, caused impairment of cell morphology, and induced excessive apoptosis in GC-2 cells, thus exhibiting an apparent germ cell toxicity.

In this study, flow cytometry results demonstrated that Ni NPs dose-dependently increased the apoptosis rate of GC-2 cells (Figure 1E), which was further validated by Western blot analysis showing upregulated expression of pro-apoptotic proteins (Bax, Caspase-3, Caspase-9) and downregulated expression of the anti-apoptotic protein Bcl-2 (Figure 4C,D). The consistency between these two orthogonal methodologies confirms that Ni NP-induced apoptosis represents a genuine biological effect rather than artifacts from the detection system. Additionally, JC-1 staining revealed reduced mitochondrial membrane potential (MMP) levels (Figure 3B,C), ATP assays indicated decreased cellular energy production (Figure 3D), and TEM observed mitochondrial cristae damage and structural fragmentation (Figure 1D). This tripartite orthogonal verification further substantiates the reliability of Ni NP-induced mitochondrial structural and functional impairments, effectively excluding potential interference artifacts from single-method measurements.

ROS, an umbrella term for oxygen-containing species, is necessary for the progression of several basic biological processes, such as the regulation of cell growth and differentiation [68]. However, excessive ROS causes the delicate balance between antioxidant defense and ROS in the body, resulting in oxidative stress, oxidative damage, mitochondrial damage, etc., consequently inducing cell apoptosis [69,70]. In the study, Ni NPs were verified to significantly increase the intracellular ROS content and MtROS, and destroy the mitochondrial morphological structure of GC-2 cells. Thus, it is speculated that Ni NPs might cause morphological deformation of mitochondria, leading to abnormal ROS accumulation and imbalance of oxidoreduction potential, subsequently inducing oxidative stress and cytotoxicity, and ultimately inducing apoptosis of germ cells.

Within cells, an electrochemical membrane potential is created by a series of proton pumps, and it is critical for maintaining the physiological function of the respiratory chain to generate ATP [71], which is the only universal energy currency in cells [72]. Sustained impairment of mitochondria results in the dysfunction of energy metabolism, thus leading to decreased ATP production, increased ROS accumulation, and destruction of MMPs [73]. Particularly, reduced MMP levels initiate the release of pro-apoptotic molecules from mitochondria, which in turn triggers the caspase cascade, ultimately facilitating the initiation and progression of apoptotic processes [73]. Caspase-9 acts as an upstream caspase that participates in triggering the activation of effector caspases, while caspase-3 functions as a commonly activated apoptotic protease tasked with the selective cleavage of numerous critical cellular proteins within the mitochondrial-mediated apoptotic signaling pathway. Currently, it is considered that Bcl-2 and Bax are members of the Bcl-2 family, which are responsible for regulating mitochondria or intrinsic apoptosis pathways [74,75,76,77], and the Bax/Bcl-2 ratio plays a vital role in regulating caspase-dependent apoptosis. In our study, treatment of GC-2 cells with Ni NPs led to a dose-dependent overgeneration of ROS, along with reduced ATP levels and MMP. These results are similar to those obtained in the study of Li and his colleagues [77]. In combination with the expression of mitochondria- and apoptosis-related proteins, our results and others suggest that Ni NPs can trigger mitochondrial dysfunction and induce excessive apoptosis in GC-2 cells through an intrinsic mitochondrial-mediated pathway, in which the mitochondrial dynamics may play a critical role in triggering and regulating sperm cell apoptosis.

### 3.2. Regulation of Mitochondria by Mdivi-1 on the Reproductive Toxicity of Ni NPs

#### 3.2.1. Determination of Optimal Conditions for Mdivi-1

**Figure 5:**
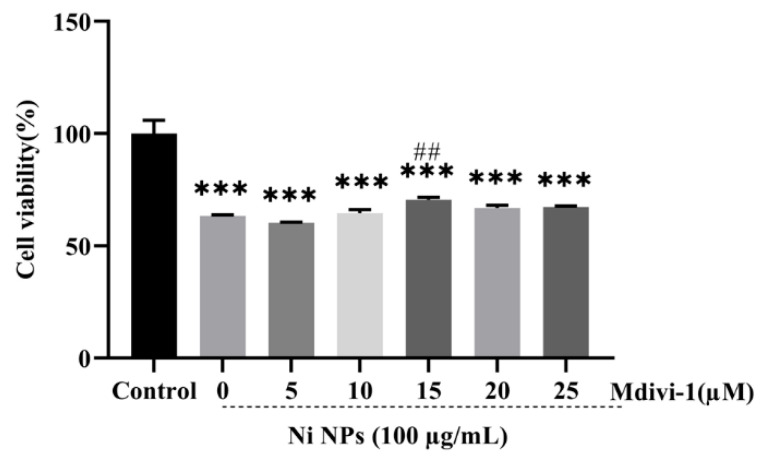
Determination of optimal conditions for Mdivi-1. Notes: *** *p* < 0.001, compared to the control group; ^##^ *p* < 0.01, compared to other Ni NP groups.

First, the optimal intervention concentration of Mdivi-1 was determined by detecting cell viability to identify the most suitable concentration of this inhibitor (Figure 5). Compared to the control group, additional Mdivi-1 inhibited the cell proliferation of GC-2 cells (*p* < 0.001), but the cell viability was higher in the 15 μM Mdivi-1 + 100 μg/mL Ni NPs group than that in other Mdivi-1 groups (*p* < 0.01). It was therefore confirmed that 15 μM Mdivi-1 was the optimal condition for the subsequent experiments.

#### 3.2.2. Effect of Mdivi-1 on Cell Apoptosis Caused by Ni NPs

**Figure 6:**
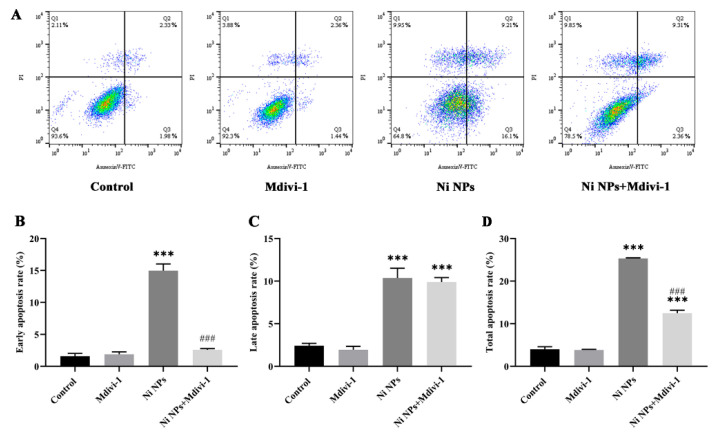
Effects of Mdivi-1 on Ni NP-induced cell apoptosis in GC-2 cells. Notes: (**A**) Dot plot images and histogram of apoptosis in GC-2 cells after different exposures for 24 h. Q1, necrotic cells; Q2, late apoptosis; Q3, early apoptosis; Q4, normal cells. (**B**) Early apoptosis rate. (**C**) Late apoptosis rate. (**D**) Total apoptosis rate. *** *p* < 0.001, compared to the control group; ^###^ *p* < 0.001, compared to the corresponding Ni NP groups.

As shown in Figure 6, compared to the control group, the early, late, and total apoptosis rates of GC-2 cells in the Ni NP groups increased significantly (*p* < 0.05). Conversely, the early and total apoptosis rates of GC-2 cells in the Ni NPs + Mdivi-1 group significantly decreased (*p* < 0.05) in comparison to the corresponding Ni NPs group. These results indicate that Mdivi-1 can inhibit the apoptosis of GC-2 cells induced by Ni NPs.

#### 3.2.3. Effects of Mdivi-1 on Oxidative Stress Caused by Ni NPs

**Figure 7:**
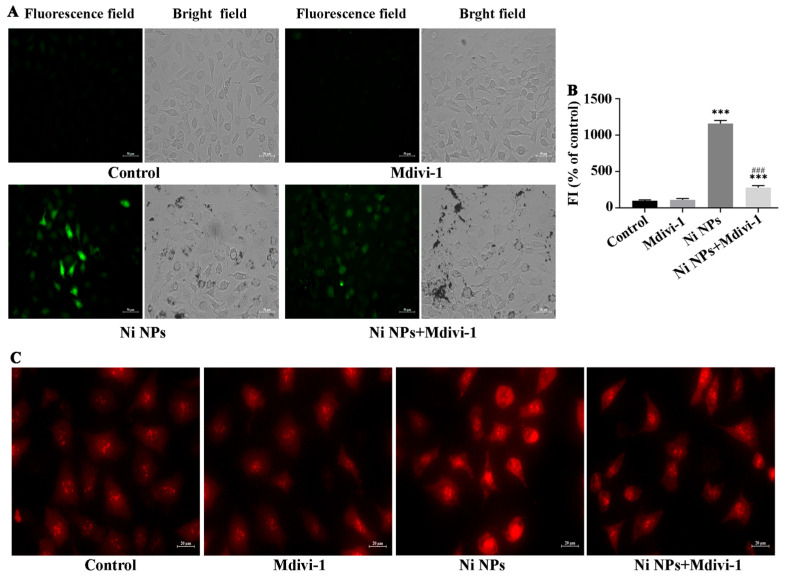
Effects of Mdivi-1 on Ni NP-induced ROS and MtROS in GC-2 cells. Notes: (**A**) Representative fluorescence images of green ROS marker (DCFH-DA) in GC-2 cells after different exposures for 24 h. (**B**) Relative green fluorescence intensity of GC-2 cells. (**C**) Representative fluorescence images of red MtROS marker in GC-2 cells after different exposures for 24 h. *** *p* < 0.001, compared to the control group; ^###^ *p* < 0.001, compared to the corresponding Ni NP groups.

To verify the effect of Ni NPs on cell oxidative stress, we added Mdivi-1, and we found that the green fluorescence intensity of ROS marker in both the Ni NP groups and the Ni NPs + Mdivi-1 group was significantly higher than that of the control group (*p* < 0.05) (Figure 7A,B). Notably, compared to the Ni NPs group, the green fluorescence intensity of ROS marker in the Ni NPs + Mdivi-1 group significantly decreased (*p* < 0.05) (Figure 7A,B). Similarly, the red fluorescence intensity of MtROS in the Ni NPs group was significantly higher than that in the control group, and that in the Ni NPs + Mdivi-1 group was significantly lower than that in the Ni NPs group. The above-mentioned results indicate that Mdivi-1 can alleviate Ni NP-induced production and accumulation of ROS and MtROS in GC-2 cells.

#### 3.2.4. Effects of Mdivi-1 on Mitochondrial Structure and Function Caused by Ni NPs

**Figure 8:**
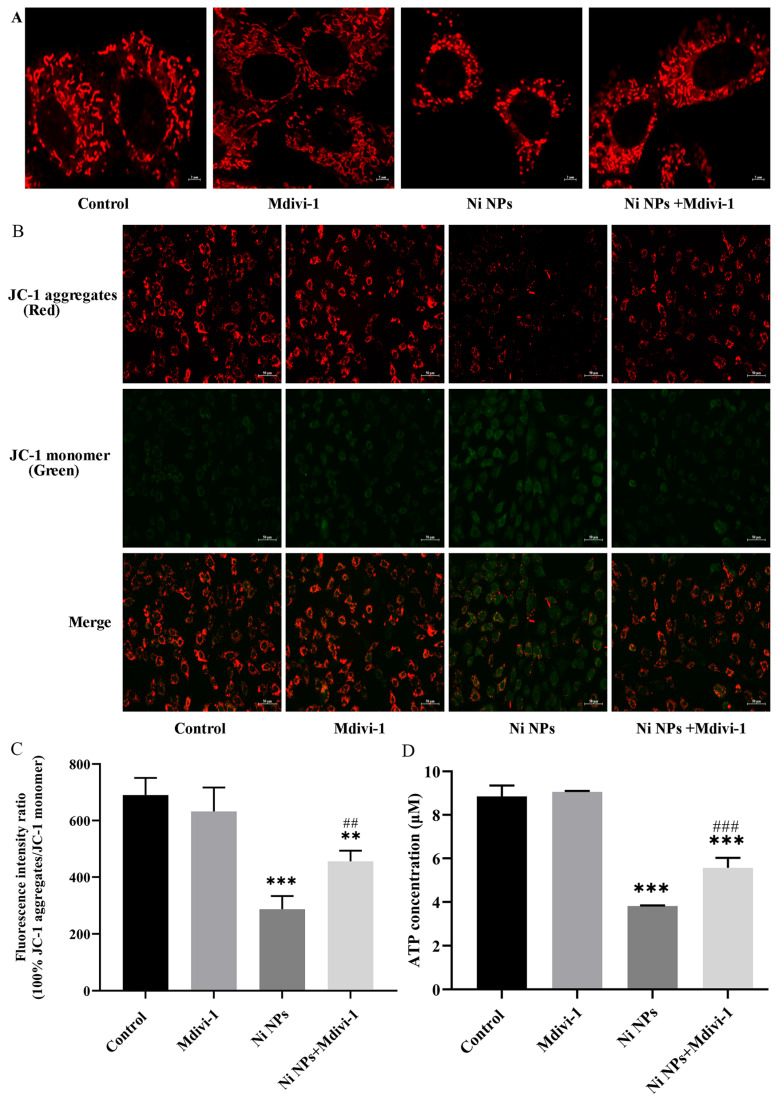
Effects of Mdivi-1 on mitochondrial structure, MMP, and ATP Ni NP-induced in GC-2 cells. Notes: (**A**) Representative mitochondrial morphology of GC-2 cells after different exposures for 24 h. (**B**) Representative fluorescence images of the MMP marker (JC-1) in GC-2 cells after different exposures for 24 h. (**C**) Relative fluorescence intensity of GC-2 cells. (**D**) ATP content of GC-2 cells after different exposures for 24 h. ** *p* < 0.01, *** *p* < 0.001, compared to the control group; ^##^ *p* < 0.01, ^###^ *p* < 0.001, compared to the corresponding Ni NP groups.

Compared to the control group, the mitochondrial network was disrupted with fragmented mitochondria in the Ni NP groups. Notably, the network structure of the mitochondria in the Ni NPs + Mdivi-1 group was improved, and the morphology gradually became normal rod and tube shapes compared to the Ni NP groups. As such, it can be inferred that Mdivi-1 could ameliorate the mitochondrial damage induced by Ni NPs, thereby inhibiting the mitochondrial division promoted by Ni NPs (Figure 8A). Similarly, intracellular MMP levels were significantly lower in GC-2 cells in both Ni NP groups and Ni NPs + Mdivi-1 group compared to the control group (*p* < 0.05) (Figure 8B,C). Interestingly, the green fluorescence of the GC-2 cells became weaker, and the red fluorescence grew stronger in the Ni NPs + Mdivi-1 compared to the Ni NP groups, indicating that the level of MMP was significantly improved (*p* < 0.05). These results suggest that Mdivi-1 could inhibit the downregulation of MMP levels in GC-2 cells induced by Ni NPs. In addition, the ATP content of the Ni NPs + Mdivi-1 group was significantly higher compared to the Ni NP groups (*p* < 0.05) (Figure 8D), implying that Mdivi-1 can alleviate the reduction in ATP content caused by Ni NPs.

#### 3.2.5. Effects of Mdivi-1 on the Expression of Mitochondria- and Apoptosis-Related Proteins Caused by Ni NPs

**Figure 9:**
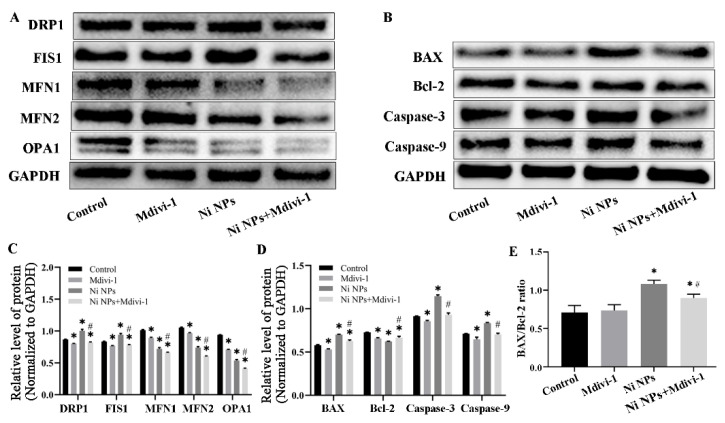
Effects of Mdivi-1 on the expression of Ni NP-induced mitochondria and apoptosis-related proteins in GC-2 cells. Notes: (**A**) Representative blot of mitochondria-related proteins in GC-2 cells after different exposures for 24 h. (**B**) Relative expression levels of the mitochondria-related proteins. (**C**) Representative blot of apoptotic proteins in GC-2 cells after different exposures for 24 h. (**D**) Relative expression levels of the apoptotic proteins. (**E**) The ratio of Bax/Bcl-2 in GC-2 cells for different exposures. * *p* < 0.05, compared to the control group; ^#^ *p* < 0.05, compared to the Ni NP groups.

Compared to the control group, the relative expression level of cellular target proteins decreased significantly in the Mdivi-1 group (*p* < 0.05). In addition, in the Ni NP-treated groups, the relative expression levels of Drp1, Fis1, Bax, Caspase-3, and Caspase-9 proteins were markedly elevated, whereas the expression of Mfn1, Mfn2, Opa1, and Bcl-2 proteins was reduced—ultimately resulting in an increased Bax/Bcl-2 ratio. (*p* < 0.05). Of note, compared to the Ni NPs group, the relative expression levels of Drp1, Fis1, Mfn1, Mfn2, Opa1, Bax, Caspase-3, and Caspase-9 proteins clearly decreased in the Ni NPs + Mdivi-1 group, and the relative expression level of Bcl-2 protein increased significantly, causing a decreased Bax/Bcl-2 ratio (*p* < 0.05). All these results demonstrate that Mdivi-1 can reverse the expression of Ni NP-induced apoptosis-related proteins in GC-2 cells, which may be related to the inhibition of enhanced mitochondrial division (see Figure 9).

Subsequently, Mdivi-1—a well-recognized inhibitor of mitochondrial fission—was introduced as a therapeutic intervention to counteract Ni NP-induced cytotoxicity in GC-2 cells, while also exploring the role of mitochondrial fission in mediating male reproductive toxicity. Our findings demonstrated that Mdivi-1 effectively mitigated several Ni NPs-triggered cellular impairments: it reversed the decline in cell viability, reduced the elevated apoptosis rate, attenuated the accumulation of both total ROS and MtROS, and restored the decreased levels of ATP and MMP. Collectively, these results confirm that Mdivi-1 is capable of alleviating Ni NP-induced cytotoxicity and the associated impairment of mitochondrial function.

Normally, mitochondrial division produces more fragmented mitochondria, and mitochondrial fusion promotes the joining of different mitochondria to produce elongated mitochondria. The present study demonstrated that the mitochondrial morphology of GC-2 cells in the Ni NPs group was significantly fragmented and dotted, while the mitochondria in the Ni NPs + Mdivi-1 group were longer and formed a more obvious network. This means that Mdivi-1 can ameliorate the Ni NP-induced mitochondrial morphological damage in germ cells by promoting the dynamic balance of mitochondrial division or fission. In addition, our present study also found that Mdivi-1 could inhibit the overexpression of division Drp1 and Fis1 proteins caused by Ni NPs, indicating that Mdivi-1 does inhibit mitochondrial division. After germ cells were exposed to Ni NPs, the overexpression of apoptotic protein Bax, protein Caspase-3, and protein Caspase-9 was alleviated by additional Mdivi-1. These results suggest that Ni NPs can disrupt mitochondrial structure and function, promote mitochondrial division, disrupt the dynamic balance, and then lead to apoptosis, implying that promoting Drp1-mediated mitochondrial division plays a key role in the toxicity of Ni NPs.

### 3.3. Effects of Low Expression of Dnm1l on the Reproductive Toxicity of Ni NPs by Regulating Mitochondrial Division

#### 3.3.1. Determination of Optimal Conditions for Dnm1l

**Figure 10:**
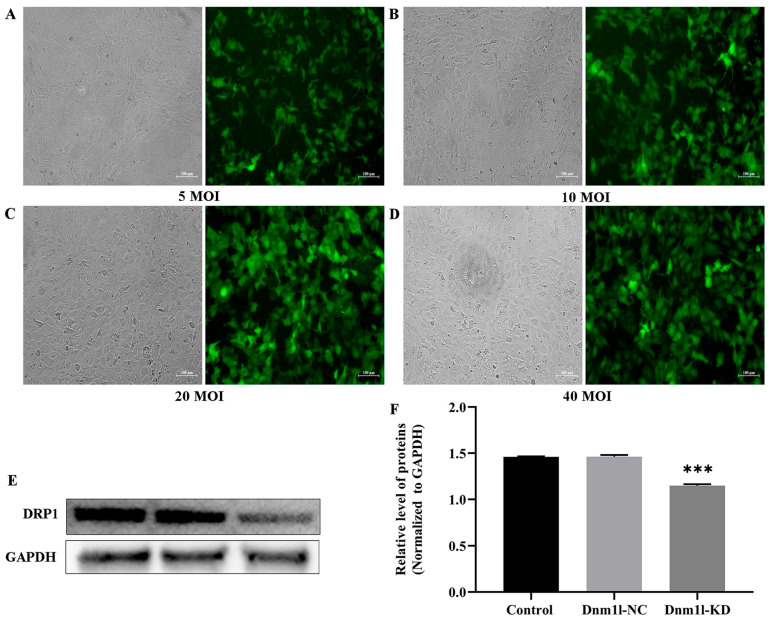
Determination of the best experimental conditions for Dnm1l. Notes: (**A**–**D**) Lentiviral infection of GC-2 cells with different MOI values for 24 h. (**E**) Representative blot of Drp1 protein in GC-2 cells after different exposures for 24 h. (**F**) Relative expression level of Drp1 protein. *** *p* < 0.001, compared to the control group.

As shown in Figure 10A–D, after GC-2 cells were infected with different MOI values of lentivirus for 24 h, the proportion of fluorescent cells in the 20 MOI group was similar to the 40 MOI group (Figure 10C,D). Notably, the green fluorescence intensity was the highest in GC-2 cells infected with 20 MOI of lentivirus (Figure 10C). Although the expression level of Drp1 in GC-2 cells of DNM1L-NC was similar to that without lentivirus infection, a lower expression level of Drp1 protein in DNM1L-KD GC-2 cells was detected, and there was a significant difference compared to control groups (*p* < 0.05) (Figure 10E,F). Therefore, we believe that 20 MOI of lentivirus is the best condition to infect GC-2 cells for Dnm1l low expression, and the stably transfected cell lines of DNM1L-KD and DNM1L-NC of GC-2 cells were screened for subsequent experimental study.

#### 3.3.2. Effects of Low Expression of Dnm1l on the Cytotoxicity Caused by Ni NPs

To further reverse verify the cytotoxicity of Ni NPs on germ cells, GC-2 cells with low expression of Dnm1l were used in the experiment to further explore the changes in apoptosis, oxidative stress, mitochondrial structure, and function, mitochondria- and apoptosis-related proteins induced by Ni NPs.

**Figure 11:**
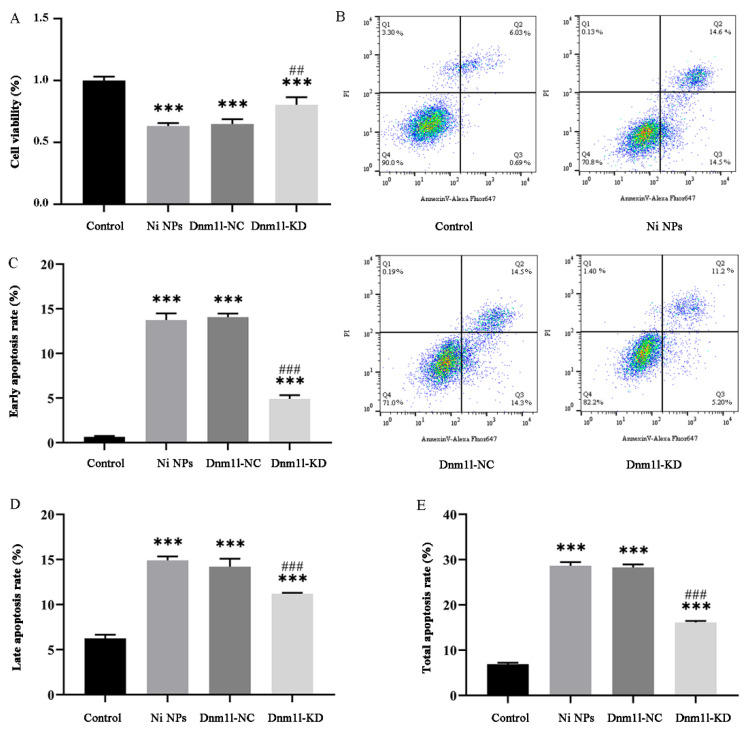
Effect of low expression of Dnm1l on Ni NP-induced cell viability and apoptosis of GC-2 cells. Notes: (**A**) Cell viability of GC-2 cells after different exposures for 24 h. (**B**–**E**) Dot plot images (**B**) and histogram (**C**–**E**) of apoptosis in GC-2 cells after different exposures for 24 h. Q1, necrotic cells; Q2, late apoptosis; Q3, early apoptosis; Q4, normal cells. (**C**) Early apoptosis rate. (**D**) Late apoptosis rate. (**E**), Total apoptosis rate. *** *p* < 0.001, compared to the control group. ^##^ *p* < 0.01, ^###^ *p* < 0.001, compared to the corresponding Ni NP groups.

After exposure to Ni NPs, the cell viability of GC-2 cells transfected with or without lentivirus infection was lower, and the cell apoptosis rate was higher compared to the control group (*p* < 0.05). Notably, the cell viability was higher in the Dnm1l-KD group than that in the corresponding Ni NP groups (*p* < 0.05) (Figure 11A). Similarly, the early, late, and total apoptosis rates were lower than those in the corresponding Ni NP groups (*p* < 0.05) (Figure 11B–E). All these results suggest that Dnm1l-KD can significantly alleviate the effect of Ni NP-induced cell viability and cell apoptosis.

#### 3.3.3. Effects of Low Expression of Dnm1l on Oxidative Stress Caused by Ni NPs

**Figure 12:**
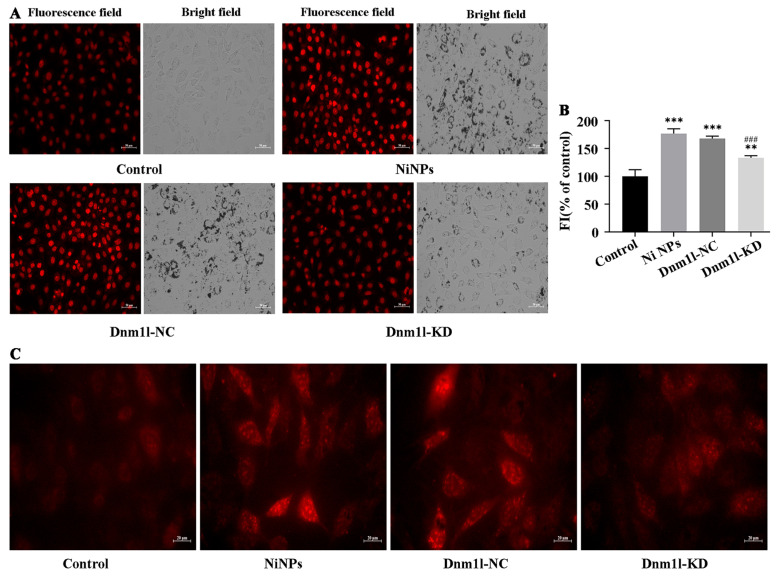
Effect of low expression of Dnm1l on Ni NP-induced ROS and MtROS in GC-2 cells. Notes: (**A**) Representative fluorescence images of green ROS marker (DHE) in GC-2 cells after different exposures for 24 h. (**B**) Relative red fluorescence intensity (FI) of GC-2 cells. (**C**) Representative fluorescence images of red MtROS marker in GC-2 cells after different exposures for 24 h. ** *p* < 0.01, *** *p* < 0.001, compared to the control group; ^###^ *p* < 0.001, compared to the corresponding Ni NP groups.

After exposure for 24 h, the intracellular red fluorescence in the Ni NPs group, Ni NPs + Dnm1l-NC group, and Ni NPs + Dnm1l-KD group became brighter, and the fluorescence intensity was significantly higher than that in the control group (*p* < 0.05) (Figure 12A,B). In addition, compared with the control group (Figure 12C), the red fluorescence intensity of GC-2 cells was similar to that of the Ni NPs + Dnm1l-KD group (Figure 12C), but it was significantly lower in comparison to the Ni NP groups (Figure 12C) and Ni NPs + Dnm1l-NC group (Figure 12C) (*p* < 0.05). These findings demonstrate that Dnm1l-KD exerts a marked inhibitory effect on the Ni NPs-elicited accumulation of intracellular ROS and MtROS.

#### 3.3.4. Effect of Low Expression of Dnm1l on the Ni NP-Induced Mitochondrial Structure and Function

**Figure 13:**
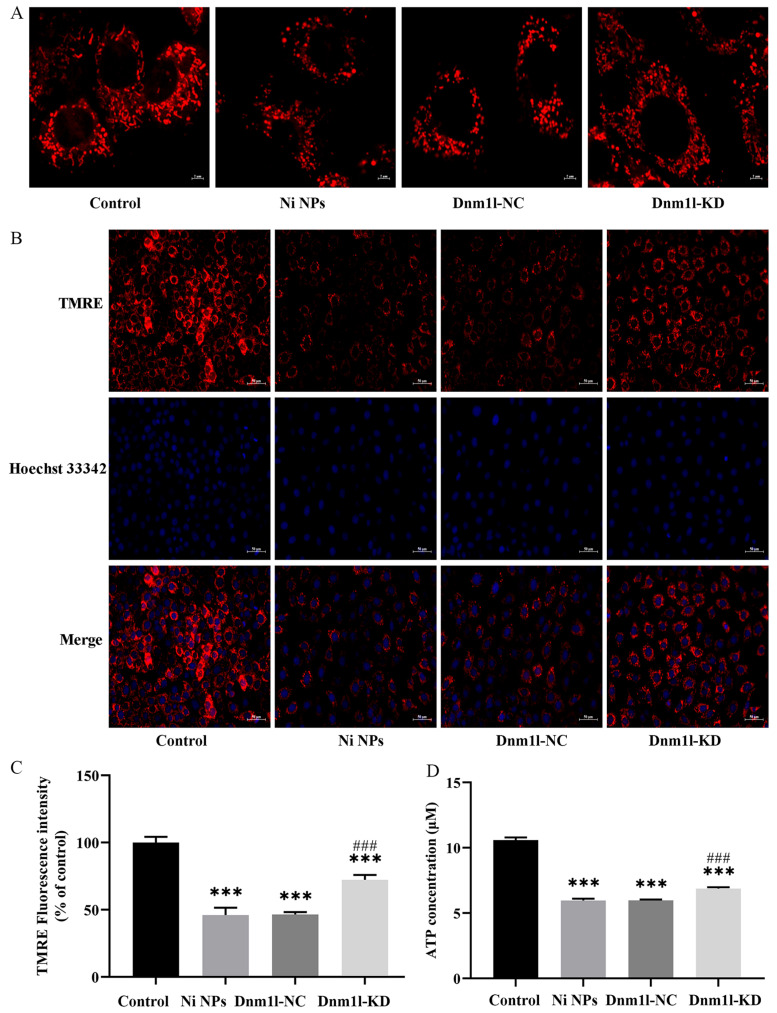
Effects of low expression of Dnm1l on the Ni NP-induced mitochondrial structure, MMP, and ATP in GC-2 cells. Notes: (**A**) Representative mitochondrial morphology of GC-2 cells after different exposures for 24 h. (**B**) Representative fluorescence images of GC-2 cells (stained with Tetramethylrhodamine and Hoechst 33342) after different exposures for 24 h. (**C**) Relative fluorescence intensity of GC-2 cells. (**D**) ATP content of GC-2 cells after different exposures for 24 h. *** *p* < 0.001, compared to the control group; ^###^ *p* < 0.001, compared to the corresponding Ni NP groups.

After exposure for 24 h, the effects of low expression of Dnm1l on Ni NP-induced mitochondrial morphology and function are shown in Figure 13. Compared to the Ni NP groups, the network structure of the mitochondria was improved, such as the gradual appearance of normal rod and tube shapes in the Ni NPs + Mdivi-1 group (Figure 13A). Similarly, the intracellular red fluorescence representing MMP levels was significantly weaker in both Ni NP groups and Ni NPs + Mdivi-1 group compared to the control group (*p* < 0.05) (Figure 13B,C). Notably, a stronger red fluorescence grew together with a weaker blue fluorescence observed in the Ni NPs + Mdivi-1 compared to the Ni NPs group. In addition, the ATP content was significantly higher in the Ni NPs + Mdivi-1 group than that in the Ni NP groups (*p* < 0.05). All these data suggest that additional Dnm1l-KD ameliorates Ni NP-induced mitochondrial fragmentation and division, alleviates Ni NP-induced downregulation of MMP level (Figure 13B,C), and significantly attenuates the decrease in ATP content induced by Ni NPs (Figure 13D).

#### 3.3.5. Effects of Low Expression of Dnm1l on the Expression of Mitochondria- and Apoptosis-Related Proteins Caused by Ni NPs

**Figure 14:**
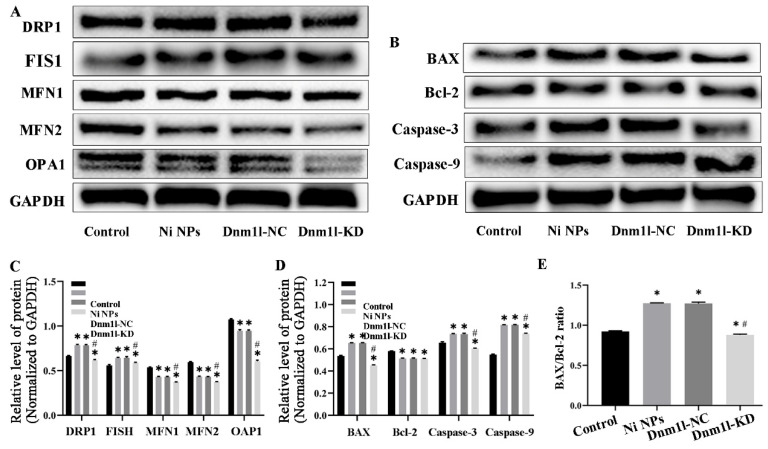
Effects of low expression of Dnm1l on the expression of Ni NP-induced mitochondria- and apoptosis-related proteins in GC-2 cells. Notes: (**A**) Representative blot of mitochondria-related proteins in GC-2 cells after different exposures for 24 h. (**B**) Relative expression levels of the mitochondria-related proteins. (**C**) Representative blot of apoptotic proteins in GC-2 cells after different exposures for 24 h. (**D**) Relative expression levels of the apoptotic proteins. (**E**) The ratio of Bax/Bcl-2 in GC-2 cells for different exposures. * *p* < 0.05, compared to the control group; ^#^ *p* < 0.05, compared to the Ni NP groups.

The Western blot assay showed the expression of mitochondria- and apoptosis-related proteins after exposure for 24 h (Figure 14). Compared to the control group, Ni NPs significantly increased the expression levels of Drp1, Fis1, Bax, Caspase-3, and Caspase-9 proteins in GC-2 cells (*p* < 0.05), whereas decreased the expression levels of Mfn1, Mfn2, Opa1 and Bcl-2 proteins (*p* < 0.05) (Figure 14A–D), accompanied with an increased Bax/Bcl-2 ratio (*p* < 0.05) (Figure 14E). Interestingly, the expression levels of Drp1, Fis1, Mfn1, Mfn2, Opa1, Bax, Caspase-3, and Caspase-9 proteins decreased significantly in the Ni NPs + Dnm1l-KD group, and the Bax/Bcl-2 ratio also apparently decreased as compared to the Ni NPs group (*p* < 0.05) (Figure 14A–D). These results indicate that additional Dnm1l-KD inhibits mitochondrial division- and apoptosis-related protein induced by Ni NPs in sperm cells.

The advances in scientific knowledge and technology have accelerated the discovery of the cellular and molecular mechanisms underlying diseases. In response to the great challenge to modern medicine originating from hereditary diseases, gene therapy has emerged as a promising treatment modality [78,79]. In 2003, China became the first country to approve gene therapy products for clinical use [80]. After two decades of clinical trials, gene therapy has shown efficacy for a series of diseases. Gene therapy is to cure genetic diseases or tumors by knocking out damaged genes or replacing them with normal genes. Common viral vectors are used as transgenic vectors. Over the past decade, lentiviral vectors have rapidly developed into reliable and safe tools for stable gene transfer in various mammalian cells [81]. In our prior experimental work, we investigated and validated the regulation of mitochondrial fission from a pharmacotherapeutic standpoint, utilizing the inhibitor Mdivi-1 as an intervention tool. In the current study, we shifted our focus to gene therapy to explore the regulatory mechanisms underlying mitochondrial fission in the context of Ni NP-induced reproductive toxicity.

Firstly, stably transfected GC-2 cell models with low expression of Dnm1l were constructed. Then, these cell models with low expression of Dnm1l or not were exposed to Ni NPs, and the results suggested that Dnm1l-KD alleviated Ni NP-induced cytotoxicity and mitochondrial damage. Accordingly, Ni NPs caused excessive apoptosis, and promoting Drp1-mediated mitochondrial division played an indispensable role.

GC-2 cell is one of the established germ cell lines that can help us better understand the complex process of spermatogenesis, involving germ cell proliferation and differentiation in the male reproductive system [82]. Studies have suggested that Ni NPs may disrupt mitochondrial dynamics homeostasis by enhancing Drp1-mediated mitochondrial division, resulting in the disorder of spermatogenesis and consequently tremendous reproductive toxicity. This potential molecular mechanism could serve as a complement to our earlier experimental investigations, thereby facilitating a more thorough and holistic understanding of the molecular regulatory pathways that underlie Ni NP-induced reproductive toxicity.

In the study, we first analyzed that Ni NP-induced reproductive toxic effects were associated with mitochondrial division through positive research, and then confirmed that promoting Drp1-mediated mitosis participated in regulating the Ni NP-induced reproductive cell toxicity through two reverse verification methods, drug therapy, and gene therapy.

Nevertheless, our results revealed that neither pharmacotherapeutic intervention with Mdivi-1 nor gene therapy involving lentivirus-mediated Dnm1l downregulation was able to reverse the Ni NP-induced reduction in the expression of mitochondrial fusion proteins—Opa1, Mfn1, and Mfn2—in germ cells. This observation suggests that mitochondrial fission may not exert a significant regulatory role in Ni NPs-triggered excessive cellular apoptosis. Notably, this finding is consistent with the research outcomes reported by Miao et al. [83]. Nevertheless, emerging evidence indicates that while Mfn1 and Mfn2 exert distinct roles in modulating spermatogonial differentiation, the loss of either protein compromises mitochondrial function and disrupts male reproductive capacity [84]. As such, the specific role of mitochondrial fusion in mediating Ni NP-induced male reproductive toxicity remains a subject of debate.

It is important to note that GC-2 cells used in this study can partially simulate the biological characteristics of spermatocytes; however, it cannot fully replicate the complex microenvironment in vivo, such as the interactions between germ cells and Sertoli cells, or the regulatory effects of endocrine factors. Additionally, the static exposure method of Ni NPs in vitro differs from the dynamic processes of absorption, distribution, metabolism, and excretion in vivo, which may lead to discrepancies in effective dosage and duration of exposure. Furthermore, although this study has systematically explored the potential mechanisms through an in vitro cellular model, it lacks validation data from in vivo experiments. These limitations highlight the need for future research to integrate both in vitro and in vivo approaches to comprehensively assess the reproductive toxicity of Ni NPs and elucidate their underlying mechanisms.

## 4. Conclusions and Outlooks

In conclusion, this study suggests that exposure of GC-2 cells to Ni NPs induces significant mitochondrial structural and functional damage, disrupts mitochondrial dynamic balance, and specifically enhances Drp1-mediated mitochondrial fission. This process further triggers excessive accumulation of ROS/mtROS and activates the mitochondrial apoptotic pathway, ultimately leading to germ cell cytotoxicity and apoptosis. Interestingly, intervention with Mdivi-1 or low expression of lentiviral Dnm1l effectively suppressed Drp1-mediated mitochondrial fission, alleviated mitochondrial dysfunction and oxidative stress, and reduced cellular apoptosis, thereby mitigating Ni NP-induced germ cell toxicity.

The innovation of this study lies in three key aspects: First, it is the first to conclusively demonstrate that Drp1-mediated mitochondrial fission serves as the central mechanism underlying Ni NP-induced male reproductive toxicity, thereby expanding previous research perspectives focused predominantly on oxidative stress and apoptotic pathways. Second, the adoption of a dual validation strategy (pharmacological inhibition using Mdivi-1 combined with Dnm1l gene silencing) provides robust causal evidence linking Drp1-dependent mitochondrial fission to Ni NP-induced germ cell toxicity. Finally, this work identifies potential therapeutic targets for mitigating Ni NP-associated reproductive damage and proposes new directions for developing protective agents against nanomaterial-induced reproductive toxicity.

However, several limitations should be acknowledged: (i) The exclusive reliance on a single in vitro cell model (GC-2) cannot fully replicate the intricate regulatory networks of testicular physiology in vivo, necessitating validation through animal models (e.g., mice or rats) to further confirm biological relevance. (ii) The exposure conditions (dosage, duration, and route) in vitro may not accurately reflect real-world environmental or occupational exposure scenarios, particularly regarding the chronic effects of low-dose Ni NP exposure. (iii) Considering the Drp1-mediated mitochondrial fission pathway, this study does not address potential crosstalk with other reported mechanisms (e.g., oxidative stress and endocrine disruption), leaving the comprehensive regulatory network of Ni NP-induced reproductive toxicity to be fully elucidated.

In the future, the transport and metabolic processes of the absorption, distribution, and excretion of Ni NPs in vivo and the effects on human cells should be investigated. Chemical modifications of Ni NPs are also investigated to maintain their functional properties while eliminating their toxicity and making them safe for practical applications.

## Data Availability

The original contributions presented in this study are included in the article/Supplementary Material. Further inquiries can be directed to the corresponding authors.

## Supplementary Materials

The following supporting information can be downloaded at: https://www.mdpi.com/article/10.3390/nano16010034/s1, Figure S1. Characterization of nickel nanoparticles (Ni NPs). (A) SEM (scale bar = 200 nm) images. (B) TEM (scale bar = 100 nm) images. (C) Particle size distributions of Ni NPs of 5 μg/mL in saline. (D) Particle size distributions of Ni NPs of 12.5 μg/mL in saline.
