# Supplementary material for: Promoting Drp1-Mediated Mitochondrial Division in Nickel Nanoparticles-Induced Reproductive Toxicity in GC-2 Cells
Source: Nanomaterials (Basel). 2025 Dec 25;16(1):34. doi: 10.3390/nano16010034 (PMC12788166; doi:10.3390/nano16010034)
Supplement: Supplementary file 1 [file nanomaterials-16-00034-s001.zip › nanomaterials-4005947-supplementary.pdf]

Supplementary Materials

Information and characterization of Nickel nanoparticles

Nickel nanoparticles (Ni NPs) were prepared by Nano Science and Technology Co., Ltd., Kunshan-miyou, Kunshan, China. The average size of Ni NPs (Product Code: FNiN-80, Bulk density: 0.06–0.8 g/cm^3, Surface area ≥ 8 m/g^2, Black Powder, Purity: 99%) were 90 nm. The scanning electron microscope (SEM) and transmission electron microscope (TEM) images showed that Ni NPs were spherical in shape. The size distribution varied from 30 to 100 nm in diameter, the average size of Ni NPs was 90 nm, and there was slight agglomeration. In the dispersion, the particle size of Ni NPs of 5 μg/mL had a distribution from 260 to 725 nm, and peak size was about 444 nm. However, the average particle size of 12.5 μg/mL had a distribution from 400 to 879 nm, and peak size of about 522 nm. For 50 μ g/mL Ni NPs, the hydrodynamic diameter was 151 ± 8 nm (PDI = 0.15); for 100 μg/mL Ni NPs, it was 248 ± 4 nm (PDI = 0.16). The zeta potential of 50 μg/mL and 100 μg/mL Ni NPs was -3.02 ± 0.19 mV and -5.60 ± 0.42 mV.

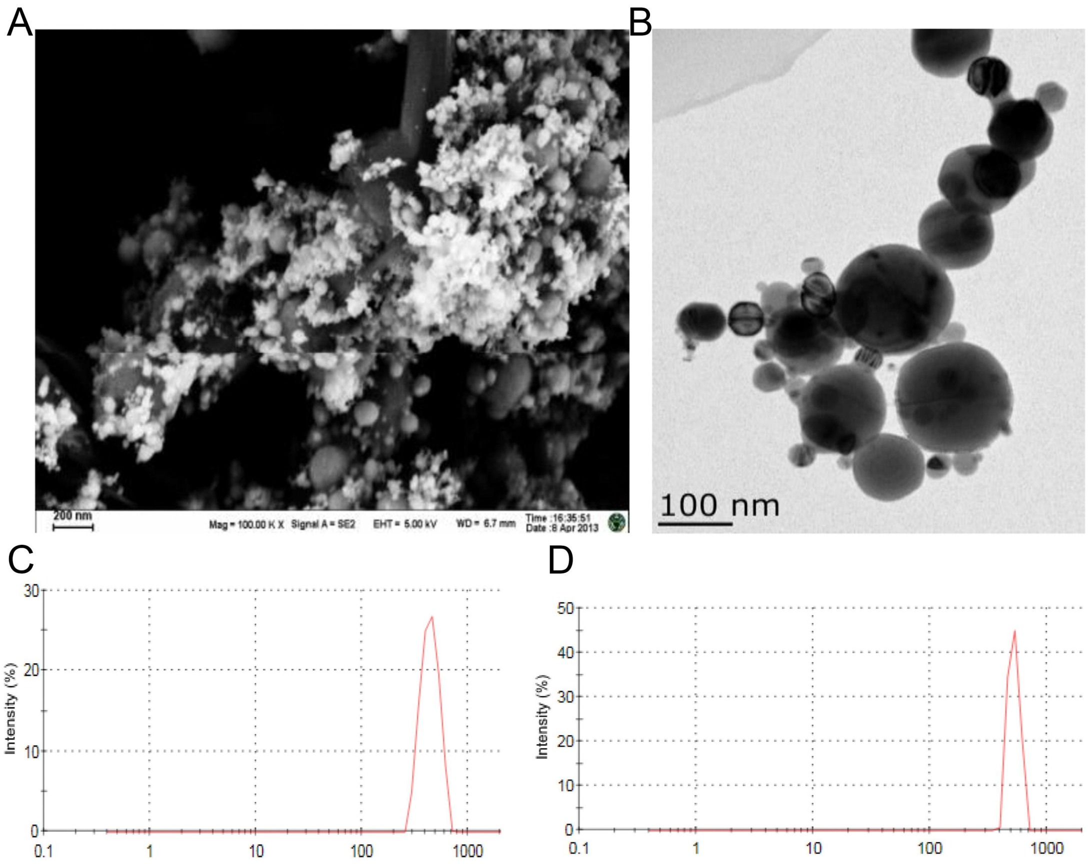

Supplementary Figure S1. Characterization of nickel nanoparticles (Ni NPs).

(A) SEM (scale bar = 200 nm) images. (B) TEM (scale bar = 100 nm) images. (C)

Particle size distributions of Ni NPs of 5 μg/mL in saline. (D) Particle size distributions of Ni NPs of 12.5 μg/mL in saline.
